# The Association of English Functional Health Literacy and the Receipt of Mammography among Hispanic Women Compared to Non-Hispanic U.S.-Born White Women

**DOI:** 10.1371/journal.pone.0164307

**Published:** 2016-10-12

**Authors:** Hajar Kadivar, Kelly M. Kenzik, Darren A. Dewalt, I-Chan Huang

**Affiliations:** 1 Department of Community Health and Family Medicine, University of Florida, Gainesville, FL; 2 Department of Health Outcomes and Policy, University of Florida, Gainesville, FL; 3 Division of Preventive Medicine, School of Medicine, University of Alabama-Birmingham, Birmingham, AL; 4 Division of General Internal Medicine, University of North Carolina at Chapel Hill, Chapel Hill, NC; 5 Department of Epidemiology and Cancer Control, St. Jude Children’s Research Hospital, Memphis, TN; National University of Singapore, SINGAPORE

## Abstract

**Background:**

Breast cancer is a leading cause of cancer death among Hispanic women in the U.S., and mammography is the recommended screening for early diagnosing and preventing breast cancer. Several barriers exist to influence mammography utilization including poor health literacy. However, it is unclear whether the effect of health literacy on mammography utilization is consistent between Hispanic women and non-Hispanic White women. The main objective of this study was to examine association between functional health literacy and the receipt of mammography among Hispanic women compared to non-Hispanic White women in the U.S.

**Methods:**

A cross-sectional design using participants engaged in the National Assessment of Adult Literacy. Study sample comprised of 4,249 Hispanic and non-Hispanic U.S.-born White women ≥ 40 years of age who completed the functional health literacy assessment. Regression analyses were performed to test the association between health literacy and receipt of mammography. Among Hispanic women, analyses considered the influence of language-preference acculturation.

**Results:**

Equal percentages of Hispanic (59.3%) and non-Hispanic White (60.6%) women received mammography. After adjusting for covariates, health literacy was positively associated with receiving mammography among U.S.-born White women (β = 0.14, p<0.001), but negatively associated with mammography among Hispanic women (β = -0.13, p<0.001). Analyses stratified by acculturation status revealed that higher health literacy was associated with lower mammography among language-preference acculturated Hispanic women (β = -0.48, p<0.001), yet an opposite result among less acculturated Hispanic women (β = 0.08, p<0.001).

**Conclusion:**

Functional health literacy has different associations with mammography depending upon ethnicity. Language-preference acculturation may explain the differing association.

## Background

Breast cancer is a leading cause of cancer death among Hispanic women within the U.S. [1]. As the Hispanic population is rapidly increasing in the U.S., it is important to assess the barriers associated with the receipt of mammography in order to determine how best to address them. Among Hispanic women, factors associated with lack of mammography utilization include inaccessible medical care (e.g., lack of health insurance), vulnerable socioeconomic status (e.g., low education), and poor knowledge and attitudes toward utilization [2, 3]. Low health literacy, defined as difficulty understanding and using health information to make health decisions, is also prevalent among Hispanic women which may affect the use of mammography [4, 5]. Previous studies have found a significant association between poor health literacy and low mammography utilization among older women (> 65 years of age) of all races/ethnicity [6, 7]. A study that stratified women by 40–64 and 65 years of age has found no association for women 40–64 years of age [7]. In contrast, other studies focusing on Hispanic women > 40 years of age found that poor health literacy was significantly associated with low mammography utilization [8, 9].

These previous studies, however, only selected certain groups of Hispanic women from regional or local communities in the U.S., and did not include a representative comparison group (e.g., non-Hispanic White women). As a result, the findings may not be generalized to the entire female Hispanic population in the U.S. Furthermore, health literacy measures used in previous studies often assessed the individual’s reading skills in a health care context (e.g., the S-TOFHLA assesses the ability to replace a missing word in a portion of health-related text, and the REALM assesses a word recognition by reading a word out lout) rather than one’s ability to use printed and written material in a relation to health-related tasks of daily life [4]. To address this issue, the concept “functional health literacy” was proposed to articulate how health literacy influences an individual on performing health care tasks and on interacting with the real health care system [10]. These limitations in previous studies pinpoint the need for additional efforts to assess the association between functional health literacy and mammography among representative Hispanic women in the U.S.

The most current and only national assessment for health literacy skills within the U.S. is the National Assessment of Adult Literacy (NAAL). The NAAL also included the assessment of participants’ cancer screening behaviors including mammography. Importantly, in 2003 all major U.S. clinical practice guidelines were in agreement with the mammography recommendation for women ≥ 40 years of age [11, 12]. Given the significance of 2003 NAAL, the aim of this study was to examine the association between functional health literacy and the receipt of mammography among Hispanic women compared to non-Hispanic U.S.-born White Women (hence referred to as White women). We hypothesized that among both Hispanic and White women, higher functional health literacy would be significantly associated with the receipt of mammography.

## Methods

### Survey Design

This study is based on a cross-sectional design of the 2003 NAAL that focused on a U.S. nationally representative household sample of more than 18,000 adults aged 16 years and older. The overall response rate before imputation was 60.1%; the response rate for the background questionnaire was 76.6%; and the response rate for the literacy assessment was 96.6% [5, 13]. The 2003 NAAL used a four-stage stratified sampling design to select study samples, including the levels of counties, census blocks, households, and individual participants. Minority area segments were oversampled to ensure the sufficient minority samples were included for analyses. Per the NAAL report, the lowest response rates from the respondents vs. the total eligible sample in the background questionnaire were among males age 30 and older in segments with high median income [5]. Trained interviewers visited the homes of participants and administered the background questionnaire orally in English or Spanish and functional literacy questionnaire in English. The functional health literacy used in the NAAL specifically assesses the concept of prose (i.e. locating, reading, understanding information in prose texts), document (i.e. locating, reading, and understanding information and commands in written instructions in documents (e.g., charts or forms)), and quantitative health literacy (i.e. locating numbers and using them to perform quantitative operations) [10]. If participants were not able to pass the screening (i.e., below the basic literacy level), the interviewers asked the questions in either English or Spanish based on the participants’ reading on English materials [5]. It has been argued that the ideal measurement of functional health literacy should assess the mismatch between an individual’s health literacy abilities and the demands of the health care system [4]. Assessing English functional health literacy can closely reflect this mismatch in the American health care system, especially in 2003 when the NAAL was administered and when translational services were more limited [14]. Total interview time was approximately 90 minutes for all participants and a $30 incentive payment was offered as compensating for their time. This study was exempt from the University of Florida Institutional Review Board.

### Study Sample

Several rules were applied to select our study sample from the overall participants in the 2003 NAAL (N = 18,102). The rules for selection included female gender, age ≥ 40 (age at which mammography screening was recommended in 2003 by medical associations such as the American Congress of Obstetricians and Gynecologists (ACOG) [11] and the American Society of Clinical Oncology (ASCO) [12]), Hispanic or White women, and a valid response to the outcome measure of mammography. A final sample contained 4,244 women; of them, 652 were Hispanic women and 3,592 were non-Hispanic White women.

### Measures

The outcome variable was self-reported mammography in the past year (yes/no). The independent variable was functional health literacy. A functional health literacy scale was used to measure three health-related tasks: clinical (e.g., filling out patient forms or reading dosing instructions), preventive (e.g., understanding how exercise prevents disease), and navigation of health care system (e.g., understanding eligibility of public health insurance programs), using every day prose and documents which did not require background knowledge [5]. Responses from three health-related tasks were combined to yield a continuous health literacy score (range: 0–500). For the purpose of interpretation, the NAAL grouped the scores into four performance levels: below basic (0–184), basic (185–225), intermediate (226–309), and proficient (310–500). However, because the AM statistical software (the only software available to analyze NAAL data) does not allow for categorical health literacy, health literacy was treated as a continuous variable in this study. Tasks related to assessing functional health literacy have been detailed elsewhere [7, 10].

NAAL is the first national assessment of the English literacy skills in Americans age 16 and older. The functional health literacy measure used in the NAAL study was built on the premise that health literacy is the ability to use printed and written information to function in society, to achieve one’s goals, and to develop one’s knowledge and potential [10]. The validity issue in the functional health literacy measure centers on content establishment through the input from experts in the Office of Disease Prevention and Health promotion within the U.S. Department of Health and Human Services. Because the NAAL uses an incomplete block-test design where each respondent answered a subset of questions, item response theory was applied to estimate literacy scores of individual domains at an aggregate level to assure the scores are comparable among groups know to be different in literacy levels such as gender and race/ethnicity [15].

Important socio-demographic characteristics of participants, including income (below or above poverty), age (40–49, 50–64, ≥ 65 years of age), and medical insurance (yes/no), were used as covariates in the statistical analyses [2, 7]. Education was not used as a covariate because it was strongly associated with health literacy. This study specifically investigated the effect of acculturation on the mammography among Hispanic women. Acculturation is an important issue to consider because women’s beliefs and attitudes regarding mammography can be changed over time as they assimilate to a new culture [16]. This study used the participants’ self-reported language preference (“Which language do you usually speak now?” English vs. Spanish) as a proxy measure for acculturation. English language preference is often used as a proxy measure for acculturation which is known as “language-preference acculturation” [17–21]. There are a variety of acculturation measures ranging from simple scales to complex multidimensional scales, and a large majority have the common thread of language use as a part of the measure. The ideal measures of acculturation would assess not only language use in a variety of context but also cultural knowledge, understanding, and identification [22]. In this study we conceptualized Hispanic women who reported primarily speaking in Spanish as less acculturated than those who reported primarily speaking in English.

### Statistical Analysis

Distributions of the binary (e.g., mammography receipt in the past year) and categorical variables (e.g., age strata) were calculated, and a chi-square test was performed to assess the statistical differences between the full Hispanic and the non-Hispanic White women samples. The association between the dependent variable (i.e., mammography) and functional health literacy with and without adjusting for covariates (i.e., medical insurance, age, and income) was analyzed using marginal maximum likelihood (MML) estimation for the full Hispanic and the non-Hispanic White women samples, respectively. MML probit regression model was specifically used in the situation of dichotomous dependent variable (i.e., mammography receipt). Additional analyses were performed by stratifying Hispanic women for the status of language-preference acculturation (i.e., English and Spanish subgroups). All analyses were implemented using AM statistical software (American Institute for Research, Beta Version 0.06.00).

## Results

Hispanic and non-Hispanic White women significantly differed by the status of medical insurance, age, income level, and functional health literacy (p’s < 0.05) (Table 1). In bivariate analyses, Hispanic women with higher functional health literacy were less likely to receive mammography services than those with lower health literacy (β = -0.07, p < 0.05) (Table 2). In contrast, non-Hispanic White women with higher functional health literacy were more likely to receive mammography services than those with lower health literacy (β = 0.08, p < 0.001). Multivariate regression analyses controlling for covariates found that higher functional health literacy remained to be significantly associated with less mammography uses among Hispanic women (β = -0.13, p < 0.001), while significantly associated with more mammography uses among non-White women (β = 0.14, p < 0.001) (Table 3).

**Table 1:** Sample characteristics by ethnicity and language-preference acculturation.

	Hispanic Women (N = 652)	U.S.-born White Women (N = 3,592)		Hispanic Women-English (N = 289)	Hispanic Women-Spanish (N = 363)	
	Weighted %	Weighted %	p-value	Weighted %	Weighted %	p-value
**Mammography**			0.754			0.35
No	40.7	39.4		43.1	38.7	
Yes	59.3	60.6		56.9	61.3	
**Health Literacy** *			**< 0.001**			**0.001**
Below Basic	52.8	10.1		16.8	82.1	
Basic	21.1	20.6		35.6	13.8	
Intermediate/ Proficient	26.2	69.4		47.5	4.2	
**Age**			**< 0.001**			0.30
40–49	50.0	32.7		53.7	46.6	
50–64	32.0	36.6		31.2	32.8	
65+	18.0	30.6		15.1	20.6	
**Income**			**< 0.001**			**0.003**
Below poverty	35.3	10.9		25.1	43.6	
Above poverty	64.7	89.1		74.9	56.4	
**Medical Insurance**			**< 0.001**			**< 0.001**
No	32.0	8.7		19.0	41.7	
Yes	68.0	91.3		81.0	58.3	

* Comparison of mean health literacy scores

**Table 2:** Bivariate analyses for the associations of socio-demographic and health literacy factors with mammography by ethnicity and language-preference acculturation.

	Hispanic Women (N = 652)	U.S.-Born White Women (N = 3,592)	Hispanic Women-English (N = 289)	Hispanic Women-Spanish (N = 363)
	β Estimate (SE)	β Estimate (SE)	β Estimate (SE)	β Estimate (SE)
**Medical Insurance (Ref = No)**				
Yes	0.441 (0.146) **	0.635 (0.110) ***	0.346 (0.302)	0.616 (0.167) **
**Age (Ref = 40–49)**				
50–64	0.209 (0.138)	0.267 (0.070) ***	0.634 (0.175) **	-0.156 (0.176)
65+	0.620 (0.155) ***	0.318 (0.084) ***	0.928 (0.24) ***	0.346 (0.200)
**Income (Ref = Below Poverty)**				
Above poverty level	0.246 (0.1) *	0.17 (0.094)	0.368 (0.159) *	0.220 (0.146)
**Health Literacy**				
Low to high scores	-0.072 (0.029) *	0.083 (0.017) ***	-0.479 (0.09) ***	0.123 (0.044) ***

*p<0.05

**p<0.01

***p<0.001

**Table 3:** Multivariate analyses for the associations of health literacy with mammography by ethnicity and language-preference acculturation.

	Hispanic Women (N = 652)	U.S.-Born White Women (N = 3,592)	Hispanic Women-English (N = 289)	Hispanic Women-Spanish (N = 363)
	β Estimate (SE)	β Estimate (SE)	β Estimate (SE)	β Estimate (SE)
**Medical Insurance (Ref = No)**				
Yes	0.457 (0.066) **	0.525 (0.040) **	0.463 (0.136) **	0.533 (0.075) **
**Age (Ref = 40–49)**				
50–64	0.174 (0.065) **	0.264 (0.029) **	0.549 (0.088) **	-0.153 (0.090)
65+	0.426 (0.072) *	0.425 (0.043) **	0.683 (0.113) **	0.197 (0.112)
**Income (Ref = Below Poverty)**				
Above Poverty Level	0.314 (0.049) **	-0.019 (0.042)	0.494 (0.077) **	0.101 (0.066)
**Health Literacy**				
Low to high scores	-0.132 (0.033) **	0.144 (0.021) **	-0.477 (0.115) **	0.078 (0.053) **

*p<0.01

**p<0.001

Analyses based on Hispanic women stratified by the status of language-preference acculturation (Table 1) found that acculturated Hispanic women (i.e., English-preferred) significantly differed from less acculturated Hispanic women (i.e., Spanish-preferred) in medical insurance enrollment and income level (p’s < 0.05). Specifically, the acculturated Hispanic women had significantly higher levels of functional health literacy than the less acculturated women (p = 0.001). Percentage of mammography use was not significantly different (56.9% vs. 61.3%, p > 0.05) between the two acculturation groups (Table 1).

Bivariate analysis found that higher functional health literacy was significantly associated with fewer mammography uses among acculturated Hispanic women (β = -0.48, p < 0.001) (Table 2). In contrast, higher health literacy was significantly associated with more mammography uses among less acculturated Hispanic women (β = 0.12, p < 0.001). Similarly, multivariate regression analysis accounting for the influence of covariates indicated that higher functional health literacy remained to be significantly associated with fewer mammography uses among acculturated Hispanic women (β = -0.48, p < 0.001), whereas significantly associated with more mammography uses among less acculturated Hispanic women (β = 0.08, p < 0.001) (Table 3).

## Discussion

Using the 2003 NAAL data, this study found that although functional health literacy was significantly associated with receipt of mammography, the direction of the association was opposite between Hispanic women and non-Hispanic White women. In contrast to our hypothesis, Hispanic women with higher functional health literacy used fewer mammography services than Hispanic women with lower health literacy. The stratification analysis further revealed that the negative association between health literacy and mammography use was only apparent for the acculturated Hispanic women (i.e., English-speaking subgroup). This study suggests that health literacy possesses differing associations with mammography which are due to the influence of ethnicity and language-preference acculturation.

Our findings in this study are in contrast to two previous studies that indicated a positive association between health literacy and mammography utilization among Hispanic women within regional communities after controlling for the acculturation [8, 9]. Similar to another study, we found that less acculturated Hispanic women were characterized with lack of health insurance and low income [3]. However, this national study also found that less acculturated Hispanic women had slightly higher, yet significant, rates of mammography utilization than the acculturated Hispanic women, which is in contrast to several previous studies [17, 23–25].

The unexpected positive association between functional literacy and mammography in less acculturated women may be explained by their unique underlying cultural beliefs that act as facilitators to the receipt of medical care. Some evidence suggests that less acculturated Hispanic women have greater beliefs in traditional family values which is associated with adherence to mammography [3]; greater belief in their personal susceptibility to and severity of breast cancer [23]; and greater trust in professionals, such as doctors who determined their health [26]. These differences may lead less acculturated Hispanic women more likely to follow physician recommendations for mammography. Furthermore, less acculturated Hispanic women, due to their lower income, may be more likely to access agencies or safety net clinics that offer free or low cost mammograms [27, 28], which may also explain why we did not find a significant association of income with mammography use in this less acculturated group. Furthermore, these agencies or safety net clinics may have better translation services than the regular clinics due to Title VI of the Civil Rights Act that requires organizations that receive federal funding to provide limited English proficiency individuals with access to language translational services [29]. As a result, it will temper the barriers among the less acculturated women. A previous study assessing the characteristics of women who utilized a mobile mammography unit serving the rural, underserved areas found that poor or incorrect knowledge about mammography was a significant predictor of adherence to mammography [30].

To our knowledge, no previous studies have found a negative association between health literacy and mammography utilization, especially among acculturated Hispanic women. Higher health literacy among acculturated women could possibly be associated with greater awareness of the potential risks of mammography such as false positives, or fears regarding radiation [31]. However, it remains unclear why the negative association between health literacy and mammography utilization was not replicated among White women. Instead, acculturation may interact with other psychosocial factors, such as anxiety and/or provider mistrust, which differ by an individual’s ethnicity and can further influence both the provider-patient interaction and patients’ follow-up for mammography [32–34]. One study found that lower acculturation was associated with a friend recommending mammography, which may act as an additional stimulus to receive mammography [8]. However, the same study did not find an association between health literacy and Hispanic women’s attitudes or beliefs about mammography [8].

This study does not diminish the importance of promoting health literacy, but does bring attention to the issues that cultural beliefs may play an important role. As a mammography recommendation is a key pre-requisite for receiving a mammogram [2], interventions to improve physicians or other healthcare providers’ communication with culturally diverse patients could help improve adherence. Among Hispanic women, fears of pain, embarrassment, and beliefs about fatalism or trauma to the breast from mammography with increased breast cancer risk can influence adherence to mammography [35–37]. Health care providers should make a greater effort to elicit and address patient fears and beliefs about mammography with greater awareness of certain culture differences. Providing cultural-sensitive patient education materials regarding mammography is one of the solutions to address barriers related to fears and beliefs.

This study has several limitations. First, despite existing guidelines for mammography, for some women, mammography may be under-utilized because of poor accessibility to health care services. Furthermore, the NAAL study relies on an individual’s self-report to collect mammography utilization data without a confirmation from medical records, which may not reflect the true receipt of mammography. Second, the NAAL did not include a comprehensive/standard acculturation measure, which could capture various components of acculturation besides language-preference. Nevertheless, language-preference acculturation has shown to be significantly associated with health behaviors in previous studies [16, 17, 19, 38]. Indeed, language-preference acculturation and language acculturation (assessed via multiple questions that assess language preference/use in a variety of contexts) are significantly associated with receipt of screening examinations compared with other components of acculturation measures (e.g., ethnic identification) and account for the greatest portion of variance of acculturation scales [39]. Third, although the NAAL assessed the use of mammography over the last year, it did not specifically enquire if the use was for diagnostic or screening purpose. Therefore, this study cannot distinguish the effect of functional health literacy on diagnosis and screening for breast cancer. Fourth, Hispanic women in the U.S., albeit primarily composed of Mexican-Americans, are not a homogenous group; Hispanic subgroups (e.g., Puerto Ricans) may possess variations in knowledge of and attitudes toward screening mammography [40, 41]. However, given the small sample size, it was impossible to break down into subgroups. Fifth, the AM statistical software does not allow for assessing interactions between health literacy and other variables (e.g., language-preference acculturation) due to a complicated stratified sampling design. To address this limitation, stratified analyses based on language-preference acculturation were performed in this study.

The NAAL study’s primary purpose was to assess English literacy; therefor, the functional health literacy measures were in English. In this context, the NAAL functional health literacy captures the essence of both functional health literacy and English literacy. The ideal measurement of health literacy should measure the mismatch between an individual’s health literacy abilities and the demands of the health care system [4]. Although, there has been increasing emphasis within the U.S. on offering universal translational services, patients still have to navigate health care systems primarily in English during the NAAL study period (early 2000’s). It is likely that English-based functional health literacy represents the health literacy mismatch [14]. However, we do not think the measurement of functional health literacy in English would lead to biased results as the negative association between health literacy and mammography receipt was among acculturated Hispanic women who preferred to speak English whose health literacy score would be less effected by language barriers.

In conclusion, functional health literacy has various effects on mammography utilization depending upon ethnicity and language-preference acculturation. Consistent with Baker’s conceptual model on the relationships of individual capacities, health literacy, and health outcomes, acculturation via its relation to other factors such as attitudes, knowledge, beliefs, and social norms also play an equally important role as health literacy in an individual’s decision to undergo mammography [4]. The interrelatedness among socio-demographic factors (e.g., ethnicity, acculturation, income, insurance, and health literacy), and how these factors interact with psychological factors (e.g., anxiety, physician mistrust) and health care systems (e.g., accessing preventive care, receiving mammography recommendation, agreeing with provider to undergo mammography, keeping mammography appointment) can influence a woman’s adherence to mammography utilization. Interventions to improve mammography rates among Hispanic women need to address barriers to mammography by especially taking both health literacy and acculturation into consideration.
